# Effect of vagus nerve stimulation against generalized seizure and status epilepticus recurrence

**DOI:** 10.3389/fneur.2023.1258854

**Published:** 2023-09-15

**Authors:** Yasushi Iimura, Hiroharu Suzuki, Takumi Mitsuhashi, Tetsuya Ueda, Kazuki Nishioka, Kou Horikoshi, Kazuki Nomura, Hidenori Sugano, Akihide Kondo

**Affiliations:** ^1^Department of Neurosurgery, Juntendo University, Tokyo, Japan; ^2^Epilepsy Center, Juntendo University Hospital, Tokyo, Japan; ^3^Sugano Neurosurgery Clinic, Tokyo, Japan

**Keywords:** vagus nerve stimulation, generalized seizure, status epilepticus, drug-resistant epilepsy, response rate

## Abstract

**Objective:**

Vagus nerve stimulation (VNS) is a palliative surgery for drug-resistant epilepsy. The two objectives of this study were to (1) determine the seizure type most responsive to VNS and (2) investigate the preventive effect on status epilepticus (SE) recurrence.

**Methods:**

We retrospectively reviewed 136 patients with drug-resistant epilepsy who underwent VNS implantation. We examined seizure outcomes at 6, 12, and 24 months following implantation of VNS as well as at the last visit to the Juntendo Epilepsy Center. Univariate analysis and multivariate logistic regression models were used to estimate the prognostic factors.

**Results:**

125 patients were followed up for at least 1 year after VNS implantation. The percentage of patients with at least a 50% reduction in seizure frequency compared with prior to VNS implantation increased over time at 6, 12, and 24 months after VNS implantation: 28, 41, and 52%, respectively. Regarding overall seizure outcomes, 70 (56%) patients responded to VNS. Of the 40 patients with a history of SE prior to VNS implantation, 27 (67%) showed no recurrence of SE. The duration of epilepsy, history of SE prior to VNS implantation and seizure type were correlated with seizure outcomes after VNS implantation in univariate analysis (*p* = 0.05, *p* < 0.01, and *p* = 0.03, respectively). In multivariate logistic regression analysis, generalized seizure was associated with VNS response [odds ratio (OR): 4.18, 95% CI: 1.13–15.5, *p* = 0.03]. A history of SE prior to VNS implantation was associated with VNS non-responders [(OR): 0.221, 95% CI: 0.097–0.503, *p* < 0.01]. The duration of epilepsy, focal to bilateral tonic–clonic seizure and epileptic spasms were not significantly associated with VNS responders (*p* = 0.07, *p* = 0.71, and *p* = 0.11, respectively).

**Conclusion:**

Following 125 patients with drug-resistant epilepsy for an average of 69 months, 56% showed at least 50% reduction in seizure frequency after VNS implantation. This study suggests that generalized seizure is the most responsive to VNS, and that VNS may reduce the risk of recurrence of SE. VNS was shown to be effective against generalized seizure and also may potentially influence the risk of further events of SE, two marker of disease treatment that can lead to improved quality of life.

## 1. Introduction

Vagus nerve stimulation (VNS) has been approved in Japan since 2010 and has been used for patients with drug-resistant epilepsy. Indications for VNS are drug-resistant epilepsy patients for whom curative surgery is difficult because the epileptic focus is difficult to detect or the epileptic focus is in an eloquent area. It is estimated that 45 to 65% of patients achieve at least a 50% reduction in seizure frequency by VNS (1–10). Previous studies have reported a variety of good VNS response factors (1–10). Although there have been reports on the efficacy of VNS for each seizure type, such as focal onset seizure, focal to bilateral tonic–clonic seizure (FBTCS), generalized seizure, and epileptic spasms, the best response candidates for seizure type still remains inconclusive (11–16).

Status epilepticus (SE) is a neurological emergency with a mortality rate of 3.45 to 22% (17, 18). The prevention of SE recurrence is important for reducing seizure burden, improving quality of life and developmental outcome in patients with drug-resistant epilepsy. The effect of VNS on SE still remains unclear. The effect of VNS on acute SE has been reported (19–21). For 38 acute-phase SE patients, seizures stopped in 28 patients in an average of 18 days after VNS implantation. However, the effect of VNS for SE remains unclear, not only in the acute-phase SE but also in the long-term prevention of SE recurrence.

The purpose of this study was to (1) determine which seizure type is most responsive to VNS and (2) investigate the protective effect on SE recurrence.

## 2. Materials and methods

### 2.1. Patient selection

Between 2010 and 2022, 136 patients with drug-resistant epilepsy who underwent VNS implantation at the Juntendo Epilepsy Center were retrospectively reviewed. All patients underwent a detailed preoperative examination at the Juntendo Epilepsy Center and were determined not to be candidates for curative epilepsy surgery. In our epilepsy center, video electroencephalography, magnetic resonance imaging, fluorodeoxyglucose-positron emission tomography, and neuropsychological testing, and, when necessary, magnetoencephalography were performed. Based on these results, a multidisciplinary conference was held to evaluate the indications for epilepsy surgery. The eligibility criteria for VNS implantation were as follows: (1) the epileptic focus could not be identified, (2) presence of multiple epileptic foci; and (3) the epileptic focus was located in an eloquent area. The implanted VNS devices implanted were either models with cardiac-based seizure detection (model AspireSR® 106, LivaNova) between 2017 and 2022 or without cardiac-based detection (models 103, 105) between 2010 and 2017. Patients who were followed up at the Juntendo Epilepsy Center for at least a year after VNS implantation were included in this study. Adjustments in antiseizure medication (ASM) and changes in VNS parameters were made in accordance with the decisions of the epileptologist.

### 2.2. Study ethics

This study was approved by the ethics committee of Juntendo University (No.16–163). Written informed consent was obtained from all the patients or their parents.

### 2.3. Seizure outcome

Outpatient charts at follow-up were used to assess seizure outcomes after VNS implantation. Postsurgical seizure outcomes were evaluated according to the McHugh classification (22). We defined the patients with class I to II as the “responder group” and the patients with class III to V as the “non-responder group” (Table 1). We collected data on seizure outcomes at 6, 12, and 24 months after VNS implantation. The overall seizure outcome was defined as the frequency of seizures at the last visit. In case of patients who underwent the epilepsy surgery after VNS implantation were considered to have the period immediately preceding the epilepsy surgery as the overall seizure outcome.

**Table 1:** Classification of seizure outcome after VNS implantation.

Class	McHugh classification	This study
1	80–100% reduction in seizure frequency	Responder
2	50–79% reduction in seizure frequency	Responder
3	<50% reduction in seizure frequency	Non-responder
4	Magnet benefit only	Non-responder
5	No improvement	Non-responder

### 2.4. Statistical analysis

All statistical analyses were performed using the SPSS Statistics 25 (IBM Corp., Chuo-ku, Tokyo, Japan). We performed the Mann–Whitney U test and Steel-Dwass test after testing for data normality using the F test. The chi-squared test or Fisher’s exact test was used to compare the categorical variables. Statistical significance was set at *p* value <0.05. Univariate analysis and multivariate logistic regression models were used to analyze the correlations between the seizure outcomes and the clinical characteristics.

## 3. Results

### 3.1. Clinical profiles

A total of 136 patients who underwent a primary VNS implantation between 2010 and 2022 at the Juntendo Epilepsy Center. Eleven patients were excluded because of insufficient follow-up and unavailable data (*n* = 8), removal less than 1 month after implantation due to infection (*n* = 2), or implantation impossible due to cardiac arrest caused by intraoperative trial stimulation (*n* = 1). Table 2 summarizes the clinical profiles of 125 patients (60 male, 65 female) who met the inclusion criteria enrolled in this study. 40 patients (32%) had history of SE prior to VNS implantation. The most common etiology of epilepsy was structural (*n* = 57, 45%), followed by genetic (*n* = 27, 22%), unknown (*n* = 25, 20%), and infectious (*n* = 16, 13%). The structural group of 57 consisted of 21 patients with bilateral temporal lobe epilepsy, 17 with unilateral temporal lobe epilepsy, 10 with focal cortical dysplasia, 3 with post-stroke and ectopic gray matter, and 1 each due to trauma, tumor, or hemangioma. The 27 genetic groups consisted of 7 Lennox–Gastaut syndrome, 7 Sturge–Weber syndrome, 6 with tuberous sclerosis complex, 3 with West syndrome, and 1 case of each of dentatorubral-pallidoluysian atrophy and cardiofaciocutaneous syndrome and CHARGE syndrome and Angelman syndrome.

**Table 2:** Clinical profiles.

	*n* = 125
Gender (Male: Female)	60: 65
Age at seizure onset (years)	13.2 ± 13.5
Age at VNS (years)	29.2 ± 15.4
Duration of epilepsy (years)	16.0 ± 12.9
Duration of follow-up period (months)	69.4 ± 42.2
Model of VNS (103/105: 106)	85: 40
History of epilepsy surgery prior to VNS	53 (42%)
**Seizure type**	
Focal onset seizure	110 (88%)
Focal to bilateral tonic–clonic seizure	65 (52%)
Epileptic spasms	22 (17%)
Generalized seizure	16 (12%)
**Etiology**	
Structural	57 (45%)
genetic	27 (22%)
infectious	16 (13%)
unknown	25 (20%)
History of SE prior to VNS	40 (32%)

SE, status epilepticus.

### 3.2. Seizure outcome after VNS implantation

**Figure 1:**
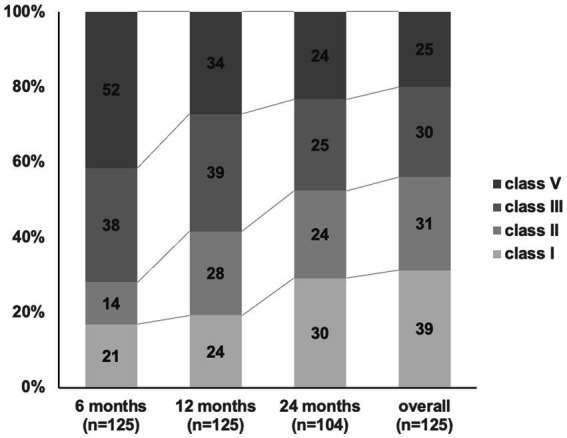
Seizure outcome after VNS implantation. The response rates (> 50% reduction, McHugh classification I-II) at 6, 12, 24 months and overall outcome after VNS implantation has increased from 28, 41, 52, and 56%.

Seizure outcomes according to McHugh classification at several follow-up points are shown in Figure 1. At 6, 12, and 24 months of follow-up, McHugh classification class I was achieved in 21 (17%), 24 (19%), and 30 (29%) patients, respectively. At 6, 12, and 24 months of follow-up, McHugh classification class II was achieved in 14 (11%), 28 (22%), and 24 (23%) patients, respectively. At 6, 12, and 24 months of follow-up, McHugh classification class III was achieved in 38 (30%), 39 (31%), and 25 (24%) patients, respectively. At 6, 12, and 24 months of follow-up, McHugh classification class V was achieved in 52 (42%), 34 (27%), and 24 (23%) patients, respectively. Overall seizure outcome, McHugh classification class I was achieved in 39 (31%), II in 31 (25%), III in 30 (24%), and V in 25 (20%). At 6, 12, and 24 months of follow-up, the number of responder patients (the total of all patients in class I and class II) was 35 (28%), 52 (42%), and 54 (52%). As the overall seizure outcome, the number of responder patients was 70 (56%).

Of the 40 patients with a history of SE before VNS implantation, 27 (67%) showed no recurrence of SE after VNS implantation. SE did not appear after VNS implantation in 83 of the 85 (98%) patients without a history of SE prior to VNS implantation.

### 3.3. Predictors of VNS responder

Table 3 shows that the duration of epilepsy, history of SE prior to VNS implantation, and seizure semiology were associated with seizure outcome after VNS implantation in the univariate analysis (*p* = 0.05, *p* < 0.01, *p* = 0.03, respectively). In the multivariate logistic regression analysis, Generalized seizure was associated with VNS response [odds ratio (OR), 4.18; 95% CI: 1.13–15.5, *p* = 0.03] (Table 4). A history of SE prior to VNS implantation was associated with non-responders to VNS (OR: 0.221, 95% CI: 0.097–0.503, *p* < 0.01). Duration of epilepsy, FBTCS and epileptic spasms were not significantly associated with VNS responders (*p* = 0.07, *p* = 0.71, and *p* = 0.11, respectively).

**Table 3:** Univariate analysis.

	**Responder (*n* = 70)**	**Non-responder (*n* = 55)**	***p* value**
Gender (Male: Female)	32: 38	29: 26	0.27
Age at seizure onset (years)	14.7 ± 13.4	11.3 ± 13.6	0.09
Age at VNS (years)	29.0 ± 14.2	29.5 ± 16.9	0.43
Duration of epilepsy (years)	14.4 ± 12.1	18.2 ± 13.7	0.05*
Duration of follow-up period (months)	73.6 ± 42.8	64.1 ± 41.2	0.11
Model of VNS (103/105: 106)	47: 23	38: 17	0.49
History of epilepsy surgery prior to VNS	25	28	0.06
Seizure type			0.03*
Focal onset seizure	56	54	
Focal to bilateral tonic–clonic seizure	35	30	
Epileptic spasms	7	15	
Generalized seizure	13	3	
Etiology			ns
structural	32	25	
genetic	12	15	
infectious	9	7	
unknown	17	8	
History of SE prior to VNS	13	27	<0.01*

SE, status epilepticus; ns, not significant.

**Table 4:** Multivariate logistic regression analysis.

	*p* value	OR	95%CI
Duration of epilepsy (years)	0.07	0.97	0.94–1.002
**Seizure type**			
Focal onset seizure	Ref		
Focal to bilateral tonic–clonic seizure	0.71	1.125	0.61–2.08
Epileptic spasms	0.11	0.45	0.17–1.19
Generalized seizure	0.03*	4.18	1.13–15.5
History of SE	<0.01*	0.221	0.097–0.503

SE, status epilepticus; Ref, reference category; OR, odds ratio; CI, confidence interval.

## 4. Discussion

### 4.1. VNS for generalized seizure

This study demonstrated the preventive effects of VNS against generalized seizure. This positive outcome in patients with generalized seizure was consistent with previous research (23). Patients with generalized seizures achieving *a* > 50% reduction in seizure frequency 1 and 2 years after VNS implantation were 46 and 49%, respectively. On the other hands, focal seizures are more likely to respond to VNS than generalized seizure (24). Although it is still controversial which type of seizure VNS is effective for, involvement of the thalamus in seizure onset suggests a mechanism for the effect of VNS on generalized seizure. According to a previous report, the thalamus is responsible for seizure onset based on a reduction in the N-acetyl aspartate/creatine ratio in the thalamus in patients with generalized seizure (25). Because VNS affect the bilateral thalamus (26), it is considered that VNS is effective against generalized seizure. We did not investigate as to which type of generalized seizure is effective because the number of patients with generalized seizure in this study was too small. Further studies are required to elucidate the mechanisms of the effectiveness of VNS against generalized seizure.

### 4.2. VNS for SE

In this study, we observed good outcomes for the recurrence of SE after VNS implantation. However, we found that the patients with a history of SE had a poor response to VNS as an overall outcome regarding the response rate of all seizure types compared to the patients without a history of SE. The outcome of VNS in SE has been reported to be favorable (27). They reported that the patients with a history of repeated episodes of SE showed improved SE and seizure frequency. VNS implantation was performed in 8 patients with episodes of SE, and 4 patients (50%) had a recurrence of SE after VNS implantation. To our knowledge, this is the first report of the preventive effect of SE in patients with episodes of SE prior to VNS implantation. However, these patients showed less than a 50% reduction in the seizure frequency of the other seizure types except SE after VNS implantation.

The mechanism of VNS against SE has not been fully elucidated. It is thought that the pathophysiological roles of *γ*-aminobutyric acid, glutamate, the inflammatory cascade, and hypoxia lead to SE (28). Moreover, the breakdown of the blood–brain barrier, inflammation, and increase may occur during the development of SE (28). This hypothesis is supported by previous studies showing some changes caused by VNS. Henry et al. showed that VNS increases cerebral blood flow, mainly in the bilateral thalamus (26). VNS-induced changes in the thalamus are significantly correlated with seizure suppression (29).

In terms of inflammatory responses, VNS was associated with a marked increase in the levels of circulating anti-inflammatory circulating cytokines (30). This cytokine response after VNS implantation may play an important role in reducing SE (31). Based on these studies, VNS may be effective against SE. It is reasonable to perform VNS implantation even if the seizure frequency, except for SE, does not improve. This study suggests a potential protective effect of VNS on SE recurrence; however, neuromodulation, such as DBS and RNS, may be an option for patients who still have other seizure types remaining.

## 5. Limitation

The present study had some limitations. This study was conducted using a retrospective survey of outpatient medical records. In addition, the assessment of seizure outcomes after VNS implantation is based on the McHugh classification, which is primarily based on seizure frequency. If the severity of the seizure is improving but the frequency of the seizure is unchanged, the McHugh classification becomes class V. Seizure outcome assessment based on classification with emphasizing the seizure severity as well as the seizure frequency may be needed in the future studies.

The next limitation is the effect of VNS on preventing the reoccurrence of SE. In this study, 27 of the 40 patients who had experienced SE prior to VNS implantation were free of SE recurrence at an average follow-up of more than 5 years. However, because SE is a rare event for most patients who experience SE, larger and longer studies are needed to determine the precise effect of VNS on the long-term risk of the recurrence of SE.

This study did not examine the relationship between seizure outcomes and ASM is not mentioned. In particular, the withdrawal of ASM may need to be considered. The present study had an average follow-up of more than 5 years and > 50% of the patients were VNS responders. In these patients, it is expected that reducing ASM can be considered, and the relationship between the seizure outcome and ASM withdrawal in patients with VNS requires further investigation.

## 6. Conclusion

A total of 125 patients with drug-resistant epilepsy were followed up for an average of 69 months, with 56% showing a good response to VNS. This study suggests that the seizure type most responsive to VNS is generalized seizure. It has also been suggested to potentially prevent the recurrence of SE in drug-resistant epilepsy patients with a history of SE prior to VNS implantation. VNS was shown to be effective against generalized seizure and also may potentially influence the risk of further events of SE, two marker of disease treatment that can lead to improved quality of life.

## Data availability statement

The original contributions presented in the study are included in the article/supplementary material, further inquiries can be directed to the corresponding author.

## Ethics statement

The studies involving humans were approved by the ethics committee of Juntendo University (No.16–163). The studies were conducted in accordance with the local legislation and institutional requirements. Written informed consent for participation in this study was provided by the participants’ legal guardians/next of kin.

## Author contributions

All authors made substantial contributions to the conception, validation, design, acquisition of data, or analysis and interpretation of data.

## Funding

This study was supported by JSPS KAKENHI (grant numbers 21K18311 and 21K09160), Health and Labor Science Research Grants on Rare and Intractable Diseases from the Ministry of Health, Labor, and Welfare, Japan (H29- nanchitou-ippan-010).

## Conflict of interest

The authors declare that the research was conducted in the absence of any commercial or financial relationships that could be construed as a potential conflict of interest. The authors declared that they were an editorial board member of Frontiers, at the time of submission. This had no impact on the peer review process and the final decision.

## Publisher’s note

All claims expressed in this article are solely those of the authors and do not necessarily represent those of their affiliated organizations, or those of the publisher, the editors and the reviewers. Any product that may be evaluated in this article, or claim that may be made by its manufacturer, is not guaranteed or endorsed by the publisher.
